# Establishment of the South Korean national antimicrobial resistance surveillance system, Kor-GLASS, in 2016

**DOI:** 10.2807/1560-7917.ES.2018.23.42.1700734

**Published:** 2018-10-18

**Authors:** Hyukmin Lee, Eun-Jeong Yoon, Dokyun Kim, Seok Hoon Jeong, Jong Hee Shin, Jeong Hwan Shin, Kyeong Seob Shin, Young Ah Kim, Young Uh, Chan Park, Kwang Jun Lee

**Affiliations:** 1 Department of Laboratory Medicine and Research Institute of Bacterial Resistance, Yonsei University College of Medicine, Seoul, Korea; 2 Department of Laboratory Medicine, Chonnam National University Medical School, Gwangju, Korea; 3 Department of Laboratory Medicine and Paik Institute for Clinical Research, Inje University College of Medicine, Busan, Korea; 4 Department of Laboratory Medicine, Chungbuk National University College of Medicine, Cheongju, Korea; 5 Department of Laboratory Medicine, National Health Insurance Service Ilsan Hospital, Goyang, Korea; 6 Department of Laboratory Medicine, Yonsei University Wonju College of Medicine, Wonju, Korea; 7 Division of Antimicrobial Resistance, National Research Institute of Health, Centers for Disease Control and Prevention, Cheongju, Korea

**Keywords:** antimicrobial resistance, surveillance, global antimicrobial resistance surveillance system, World Health Organization, bacterial collection, Kor-GLASS

## Abstract

Surveillance plays a pivotal role in overcoming antimicrobial resistance (AMR) in bacterial pathogens, and a variety of surveillance systems have been set up and employed in many countries. In 2015, the World Health Organization launched the Global Antimicrobial Resistance Surveillance System (GLASS) as a part of the global action plan to enhance national and global surveillance and research. The aims of GLASS are to foster development of national surveillance systems and to enable collection, analysis and sharing of standardised, comparable and validated data on AMR between different countries. The South Korean AMR surveillance system, Kor-GLASS, is compatible with the GLASS platform and was established in 2016 and based on the principles of representativeness, specialisation, harmonisation and localisation. In this report, we summarise principles and processes in order to share our experiences with other countries planning to establish a national AMR surveillance system. The pilot operation of Kor-GLASS allowed us to understand the national burden of specific infectious diseases and the status of bacterial AMR. Issues pertaining to high costs and labour-intensive operation were raised during the pilot, and improvements are being made.

## Background

Antimicrobial resistance (AMR) in bacterial pathogens has become one of the most important threats to public health around the world, resulting in high morbidity and mortality, prolonged hospitalisation and increased medical expenses [1]. In 2014, O’Neill et al. estimated that AMR will cause 10 million deaths and 100.2 trillion USD in losses per year in world gross domestic product (GDP) by 2050 [2]. The World Health Organization (WHO) presented the global action plan on AMR in 2015 [3], consisting of five strategic objectives: (i) to improve awareness and understanding of AMR; (ii) to strengthen knowledge through surveillance and research; (iii) to reduce the incidence of infection; (iv) to optimise the use of antimicrobial agents; and (v) to develop an economic case for sustainable investment that takes into account the needs of all countries and increases investment in new medicines, diagnostic tools, vaccines and other interventions.

Surveillance is one of the pivotal components necessary to overcome AMR, and a variety of surveillance systems for humans have been set up in many countries [1]. A representative example is the European Antimicrobial Resistance Surveillance Network (EARS-Net) coordinated by the European Centre for Disease Control and Prevention [4]. The network collects harmonised AMR data about invasive isolates from member countries’ national AMR surveillance systems. However, there are many differences among surveillance systems in terms of target microorganisms, target antimicrobial agents, monitoring methods, reporting methods, monitoring periods and so on [1]. These differences make it difficult to compare and interpret AMR results from each surveillance system and to establish appropriate policies and plans to control AMR. In 2015, WHO launched the Global Antimicrobial Resistance Surveillance System (GLASS) [5] which allows ascertainment of the most frequent type of AMR bacterial infections, the age- and sex-structures of infections, infection types (community origin (CO), hospital origin (HO)), and therefore provides better understanding of the impact of AMR on human health.

The Korea Centers for Disease Control and Prevention (KCDC) operated a nationwide AMR surveillance system (South Korean Antimicrobial Resistance Monitoring System, KARMS) between 2002 and 2015 [6-8]. The system collected laboratory data to monitor AMR and to detect emerging resistance from sentinel hospitals. However, issues regarding data reliability were raised for KARMS, resulting from differences in antimicrobial susceptibility testing methods and interpretation breakpoints by hospital. Insufficient deduplication of clinical isolates and data was also a problem. The Ministry of Health and Welfare of South Korea released a National Action Plan in 2016 [9] including the establishment of a new national AMR surveillance system, Kor-GLASS, which is compatible with the GLASS platform. This new system is based on the collection of non-duplicate clinical isolates and data by specimen from sentinel hospitals. The collected isolates are characterised in an analysis centre with a standardised method allowing harmonised data.

South Korea is currently experiencing challenges with AMR. The most recent KARMS report stated that meticillin-resistant *Staphylococcus aureus* is common (ca. 66% of *S. aureus*), and among tested *Acinetobacter baumannii* and *Pseudomonas aeruginosa* isolates collected from 16 general hospitals (intensive care and general wards) in 2015, 85% and 35% tested were imipenem-resistant positive, respectively [8]. The establishment of Kor-GLASS is one of the ways in which South Korea is tackling the problem. In this article, we summarise principles and processes established for Kor-GLASS in order to share our experiences with other countries planning to establish a national AMR surveillance system.

## Setting and structure of Kor-GLASS

South Korea is an urbanised country with a population of 51.02 million and GDP per capita of 23,306 EUR (27,097 USD) in 2015 [10], making South Korea’s GDP the 30th highest in the world. The medical system is composed mainly of private medical institutions, and the proportion of hospital beds in public medical institutions (10.4%) is low. Hospitals are categorised as primary (n = 32,268), secondary (n = 301), and tertiary care (n = 43). Primary-care hospitals have outpatients only, while secondary- and tertiary-care hospitals have both outpatients and inpatients.

The structure of Kor-GLASS has been designed in accordance with the four principles of representativeness, specialisation, harmonisation and localisation. Kor-GLASS was set up and is governed by the KCDC.

## Representativeness

**Figure 1:**
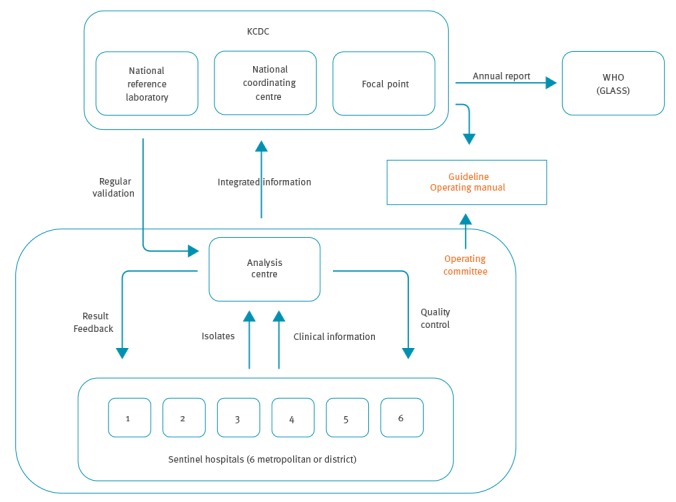
Structure and governance of the national antimicrobial resistance surveillance system Kor-GLASS, South Korea

GLASS: Global Antimicrobial Resistance Surveillance System; KCDC: Korea Centers for Disease Control and Prevention; WHO: World Health Organization.

The KCDC: (i) established the structure; (ii) funds the study; (iii) governs the system; and (iv) operates the national coordinating centre (Division of Infectious Disease Surveillance), the national focal point (Division of Healthcare-Associated Infection Control) and the national reference laboratory (Division of Antimicrobial Resistance) (Figure 1).

The system has been designed to collect and analyse complete non-duplicate clinical isolates and information from six sentinel hospitals (each with a capacity of between 655 and 1,000 beds) caring for both inpatients and outpatients (Figure 1), representing four of nine provinces and two of seven metropolitan cities in South Korea (Supplementary Figure 1). The population covered by each sentinel hospital ranges from 1.55 to 12.71 million.

## Specialisation

Kor-GLASS has operational and advisory committees consisting of members with expertise in infectious disease and clinical microbiology in South Korea. Clinical isolates and information collected through the system are scrutinised in an analysis centre with both hard- and software appropriate for AMR study. The capability of the diagnostic microbiologic laboratories of sentinel hospitals to produce accurate and reproducible data was another consideration. The sentinel hospitals were selected according to the criteria of having: (i) an occupational clinical microbiologist; (ii) a clinical microbiology laboratory certified by both the Quality Assessment programme and the External Quality Control programme in South Korea [11]; and (iii) a laboratory information system and electronic medical record service to ease epidemiological data collection.

## Harmonisation

All isolates collected are transferred to the analysis centre and assessed together for AMR pheno- and genotyping and strain typing for molecular epidemiology using harmonised testing methods, using a formatted clinical data collection system. Bacterial collection guidelines by specimen including isolation, storage and transfer are shared with sentinel hospitals, and educational programmes for laboratory personnel are held twice a year. Clinical data including infection origin (CO and HO), age, sex and admission types (outpatient, inpatient in general ward or intensive care unit) are collected for all patients for whom blood, urine, stool and urethral/cervical discharge have been cultured, regardless of culture positivity or negativity. Infection origins are categorised according to the number of hospitalisation days at the time of specimen sampling: HO, if the specimen was taken at ≥ 2 calendar days of hospitalisation including the days hospitalised in a previous healthcare facility before transfer; CO, if the specimen was taken either from an outpatient or from an inpatient of < 2 calendar days of hospitalisation. The data are further used to analyse the AMR burden in patients admitted to sentinel hospitals.

## Localisation

To reflect South Korean AMR traits, the GLASS manual has been customised as follows: (i) three additional target pathogens were included: *Enterococcus faecalis*, *E. faecium*, and *P. aeruginosa* blood isolates to monitor the glycopeptide and carbapenem resistance critical in South Korean clinical settings; (ii) further target antimicrobials to categorise multi-drug resistance were added, so in the case of *S. aureus*, for example, we tested not only cefoxitin as in the GLASS manual, but also erythromycin, clindamycin, co-trimoxazole, quinupristin-dalfopristin, mupirocin, vancomycin, teicoplanin, linezolid and tigecycline; and (iii) auxiliary genetic analysis to understand the extent of resistance determinants in South Korean clinical settings and strain typing to assess molecular epidemiology of drug-resistant clones dominant in the country.

## Operating manual for handling samples within Kor-GLASS

**Figure 2:**
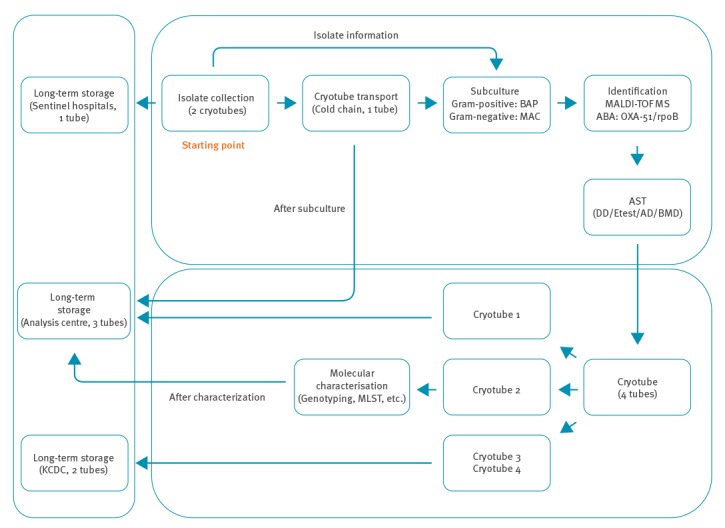
Schematic flow of collection, transfer, analysis and storage of clinical isolates in the national antimicrobial resistance surveillance system Kor-GLASS, South Korea

ABA: *Acinetobacter baumannii*; AD: agar dilution; AST: antimicrobial susceptibility testing; BAP: blood agar plate; DD: disk diffusion; KCDC: Korea Centers for Disease Control and Prevention; MAC: MacConkey agar plate; MALDI-TOF: Matrix-Assisted Laser Desorption/Ionization Time-of-Flight; MLST: multilocus sequence typing; MS: mass spectrometry.

There are four steps within Kor-GLASS: collection and transfer of clinical isolates, basic characterisation, advanced characterisation and long-term storage (Figure 2).

## Collection and transfer of clinical isolates

The target pathogens in Kor-GLASS are *S. aureus*, *Streptococcus pneumoniae, E. faecalis, E. faecium, Escherichia coli, Klebsiella pneumoniae, P. aeruginosa, A. baumannii*, and *Salmonella* species recovered from blood specimens; *Salmonella* and *Shigella* species from stool specimens; *E. coli* and *K. pneumoniae* from urine specimens; and *Neisseria gonorrhoeae* from urethral/cervical discharge specimens. All isolates of target pathogens from target specimens are collected in each sentinel hospital. Clinical isolates are recovered through enrichment culture for blood specimens, selective cultures for stool and urethral/cervical discharge specimens. For urine specimens, semi-quantitative culture is carried out to judge if the isolate meets the following criteria: (i) ≥ 10^4^ CFU/mL single-species growth of either *E. coli* or *K. pneumoniae* or (ii) ≥ 10^5^ CFU/mL of *E. coli* or *K. pneumoniae* in growth of mixed species [12]. Each isolate is inoculated into two cryotubes containing 20% (w/v) skimmed milk [13]: one for long-term storage at − 80 °C in each sentinel hospital and the other for transfer to the analysis centre. Bacterial isolate transfer is carried out twice a month, maintaining the cold chain. For the transferred clinical isolates, purity and viability are verified by sub-culture. If an isolate does not grow or has been contaminated, the sentinel hospitals are asked to re-send the isolate. Feedback is given in case of contamination and no growth to improve the performance in sentinel hospitals.

## Basic characterisation of collected isolates and reporting

Bacterial species of the collected isolates are verified using Matrix-Assisted Laser Desorption/Ionization Time-of-Flight (MALDI-TOF) mass spectrometry (Bruker Biotyper, Bruker Daltonics GmbH, Bremen, Germany) in the analysis centre. *Acinetobacter* species are identified with a species-specific PCR targeting *bla*_OXA-51_ and with *rpoB* gene sequencing [14]. Species identification discrepancy between a sentinel hospital and the analysis centre is confirmed by 16S rDNA sequencing [15] and feedback is given. Antimicrobial susceptibility is mainly determined by the disk diffusion test following the Clinical and Laboratory Standards Institute (CLSI) guidelines revised in 2016 (M100-S26) [16]. Etest, agar dilution and broth microdilution tests are also employed in cases where disk diffusion is unavailable or minimum inhibitory concentration (MIC) is needed for further analysis (Supplementary Table 1). Colistin MIC is determined by the broth microdilution method following the recommendations of the joint CLSI-EUCAST Polymyxin Breakpoints Working Group [17]. Images from disk diffusion and Etest are stored for checking data-entry mistakes. Blind cross-checks for antimicrobial susceptibility testing between the analysis centre and the national reference laboratory are also carried out on a monthly basis to validate the results. Third-party verification is made for any discrepancy and a correction is made following the recommendations of the advisory committee. Basic characterisation of the collected isolates in the analysis centre takes ca 2 months. The national focal point collects the results of basic characterisation integrated with clinical data monthly and the national coordinating centre reports Kor-GLASS’s results to WHO annually.

## Advanced characterisation of collected isolates

The analysis centre performs advanced characterisation, including AMR genotyping and strain typing, to assess the molecular epidemiological characteristics of major pathogens. AMR genotyping is done for methicillin resistance in *S. aureus*, vancomycin resistance in enterococci, third- and fourth-generation cephalosporin, carbapenem and colistin resistance in Gram-negative bacilli (Table 1). SCC*mec* typing for meticillin-resistant *S. aureus* is also performed by multiplex PCR as described previously [18]. Multilocus sequence typing (MLST) is carried out following species-specific methods and *agr* and *spa*-typing is performed for *S. aureus* as described previously [19]. Advanced characterisation of the collected isolates in the analysis centre takes an additional 2 months. The national focal point collects aggregated results monthly from the analysis centre.

**Table 1:** Molecular characterisation of AMR determinants and strain types, national antimicrobial resistance surveillance system Kor-GLASS, South Korea

Species	AMR determinants	Epidemiological markers
*Staphylococcus aureus*	• Meticillin-resistance gene PCR (*mecA*/*mecC*) • SCC*mec* typing • *agr* polymorphism typing	• MLST • *spa*-typing
*Enterococcus faecalis* *E. faecium*	• Glycopeptide-resistance gene PCR (*vanA*/*vanB*/*vanM*) • Tn*1546* analysis	• MLST • Plasmid analysis (replicon typing)
*Escherichia coli* *Klebsiella pneumoniae*	• ESBL gene PCR and sequencing (*bla*_CTX-M-1_, *bla*_CTX-M-9_, *bla*_CTX-M-2_, *bla*_CTX-M25_, *bla*_TEM_, *bla*_SHV_) • AmpC β-lactamase PCR and sequencing (*bla*_DHA_, *bla*_CMY-1_, *bla*_CMY-2_, *bla*_ACT_, *bla*_ACC_, *bla*_FOX_) • Carbapenemase gene PCR and sequencing (*bla*_KPC_, *bla*_NDM_, *bla*_OXA-48_, *bla*_VIM_, *bla*_IMP_, *bla*_GES_) • Mobile colistin resistance gene PCR (*mcr-1/-2*)	• MLST • Plasmid analysis (replicon typing)
*Pseudomonas aeruginosa*	Carbapenemase gene PCR and sequencing (*bla*_KPC_, *bla*_NDM_, *bla*_OXA-48_, *bla*_VIM_, *bla*_IMP_, *bla*_GES_)	• Class 1 integron • MLST
*Acinetobacter* spp.	• Carbapenemase PCR and sequencing (*bla*_OXA-23/-24/-51/-58_, *bla*_KPC_, *bla*_NDM_, *bla*_OXA-48_, *bla*_VIM_, *bla*_IMP_, *bla*_GES_)	• Transposon • MLST
*Salmonella* spp. *Shigella* spp.	• CTX-M ESBL PCR and sequencing (*bla*_CTX-M-1_, *bla*_CTX-M-9_, *bla*_CTX-M-2_, *bla*_CTX-M-25_) • Fluoroquinolone resistance gene PCR and sequencing (*repA, gyrA, parC*)	NA
*Neisseria gonorrhoeae*	• Cephalosporin-resistance gene analysis (*penA*) • Spectinomycin-resistance gene PCR and sequencing (16S rRNA, *rpsE*, 23S rRNA) • Azithromycin-resistance gene PCR (*ermB, ermC, ermF, mef*)	• NG-MAST • MLST

AMR: antimicrobial resistance; ESBL: extended-spectrum beta-lactamase; MLST: multilocus sequence typing; NA: not applicable; NG-MAST: *Neisseria gonorrhoeae* multi-antigen sequence typing.

## Long-term storage of collected isolates

Four cryotubes containing each isolate in 20% skimmed milk are created in the analysis centre: two for storage in the national reference laboratory for further construction of a national bank for AMR strains, one for long-term storage in the analysis centre and the other for working stock. Eventually, each isolate is kept independently at three separate sites: one in the sentinel hospital, two in the analysis centre and two in the national reference laboratory.

## Budget for Kor-GLASS

The budget consists of analysis costs and collection and transfer costs. The cost for analysis was calculated using the number of isolates collected during Kor-GLASS’s pilot in 2016, multiplied by unit cost of each test (Table 2). The average of unit cost for analysing each isolate was 57 EUR (71 USD) for blood isolates, ranging from 22 to 164 EUR (28–206 USD), depending on the species; 21 EUR (27 USD) for urine isolates and 29 EUR (36 USD) for stool isolates. The total cost for analysis of 110,911 isolates collected was 631,077 EUR (788,854 USD): 468,996 EUR (586,251 USD) for blood isolates (n = 3,523); 159,833 EUR (199,792 USD) for urine isolates (n = 7,491); and 2,248 EUR (2,811 USD) for stool isolates (n = 77). The cost for collection and transfer per sentinel hospital was 32,000 EUR (40,000 USD) and, multiplied by the six sentinel hospitals, the total cost was 192,000 EUR (240,000 USD). The total budget to run the system was 823,077 EUR (1,028,854 USD).

**Table 2:** Budget for the analysis centre of the national antimicrobial resistance surveillance system Kor-GLASS, South Korea, pilot year, May 2016 to April 2017

Specimen	Species	Number of isolates	Cost in EUR (USD)								
			Identification	Disk diffusion	Etest	Agar dilution	PCR	Genotypic sequencing	MLST	Average cost /isolate	Total
			6 (7.4) /isolate	0.8 (1) /disk	2.4 (3) /strip	16 (20) /isolate	8 (10) /reaction	8 (10) /gene	80 (100) /isolate		
**Blood**	*Staphylococcus aureus*	584	3,504 (4,380)	2,803 (3,504)	5,606 (7,008)	NT	37,376 (46,720)	NT	46,720 (58,400)	164 (206)	96,009 (120,012)
	*Streptococcus. pneumoniae*	28	168 (210)	NT	NT	448^a^ (560)^a^	NT	NT	NT	22 (28)	616 (770)
	*Enterococcus faecalis*	161	966 (1,208)	644 (805)	1,546 (1,933)	NT	2,576 (3,220)	NT	12,880 (16,100)	116 (145)	18,612 (23,266)
	*E. faecium*	217	1,302 (1,628)	1,042 (1,303)	2,083 (2,604)	NT	3,472 (4,340)	NT	17,360 (21,700)	116 (145)	25,259 (31,575)
	*Escherichia coli*	1,536	9,216 (11,520)	23,347 (29,184)	NT	246 (308)	30,259 (37,824)	9,062 (11,328)	122,880 (153,600)	127 (159)	195,010 (243,764)
	*Klebsiella pneumoniae*	597	3,582 (4,478)	8,597 (10,746)	NT	478 (598)	15,761 (19,701)	4,716 (5,895)	47,760 (59,700)	136 (169)	80,894 (101,118)
	*Salmonella* spp.	44	264 (330)	NT	NT	704^b^ (880)^b^	176 (220)	141 (176)	NT	29 (36)	1,285 (1,606)
	*Pseudomonas aeruginosa*	127	762 (953)	1,118 (1,398)	NT	406 (508)	1,829 (2,286)	305 (381)	10,160 (12,700)	115 (143)	14,580 (18,226)
	*Acinetobacter* spp.	229	1,374 (1,718)	2,564 (3,205)	NT	2,931 (3,664)	9,893 (12,366)	1,649 (2,061)	18,320 (22,900)	160 (200)	36,731 (45,914)
	Subtotal	3,523	21,138 (26,425)	40,115 (50,145)	9,235 (11,545)	5,213 (6,518)	101,342 (126,677)	15,873 (19,841)	276,080 (345,100)	133 (166)	468,996 (586,251)
**Urine**	*E. coli*	6,394	38,364 (47,955)	97,189 (121,486)	NT	1,023 (1,279)	NT	NT	NT	21 (27)	136,576 (170,720)
	*K. pneumoniae*	1,097	6,582 (8,228)	15,797 (19,746)	NT	878 (1,098)	NT	NT	NT	21 (27)	23,257 (29,072)
	Subtotal	7,491	44,946 (56,183)	112,986 (141,232)	NT	1,901 (2,377)	NT	NT	NT	21 (27)	159,833 (199,792)
**Stool**	*Salmonella* spp.	77	462 (578)	NT	NT	1,232^b^ (1,540)^b^	308 (385)	246 (308)	NT	29 (36)	2,248 (2,811)
**Total**		**11,091**	**66,546** **(83,186)**	**153,101** **(191,377)**	**9,235** **(11,545)**	**8,346** **(10,435)**	**101,650** **(127,062)**	**16,119** **(20,149)**	**276,080 (345,100)**	**57** **(71)**	**631,077** **(788,854)**

MLST: multilocus sequence typing; NT: not tested.

^a^ Costs for each test are rounded up to the nearest EUR.

^b^ Antimicrobial susceptibilities of *S. pneumoniae* and *Salmonella* species were all tested by the broth microdilution method.

Exchange rate of 1 EUR = 1.25 USD applied.

## Pilot operation of Kor-GLASS

Kor-GLASS began operation on 1 May 2016, and ca 1,000 isolates were collected each month for the first 12 months. Although operational and advisory committees took charge of designing Kor-GLASS, several operating issues were raised.

First, *Shigella* species and *N. gonorrhoeae* causing CO infections were not isolated from the sentinel hospitals. Shigellosis had been the dominant acute bacterial gastroenteritis in South Korea until the 1970s, but prevalence declined from the late 1980s and was rare by the 2010s [20]. Gonorrhoea is common in South Korea, and ca 15,000 cases are reported every year. More than 95% of patients with gonorrhoea in South Korea are diagnosed at primary care clinics [21]. Therefore, another surveillance system that included primary care clinics specialising in gonorrhoea was needed to monitor *N. gonorrhoeae* AMR. Secondly, although the sentinel hospitals operated the EMR system, it was very burdensome for laboratory personnel to collect the clinical data. Discriminating between CO and HO infections was another challenge due to difficulties in researching hospitalisation days in a previous healthcare facility before transfer. Thirdly, the analysis centre found the workload of the regular report and the characterisation of a huge collection to be very heavy. The number of laboratory personnel in the analysis centre doubled, from eight to 16, in 1 year. Finally, discrepancies between AMR phenotypes and genotypes were identified. For instance, a few Enterobacteriaceae clinical isolates exhibiting extended-spectrum beta-lactamase (ESBL) or carbapenemase phenotypes did not carry any known ESBL or carbapenemase genes, and further analysis for the novel resistance determinants was needed.

## Future plans for Kor-GLASS

Following the pilot study phase, Kor-GLASS is now expanding: (i) coverage is being enlarged by recruiting further sentinel hospitals in districts not yet covered, including hospitals for long-term care, and by adding research targets (uncovered bacterial pathogens, recently launched antimicrobials, and emerging AMR determinants); (ii) a quality control centre is being established for quality improvement; (iii) an AMR bacterial bank is being constructed to enable the use of clinical isolates for further research; (iv) helping the general public better understand the antimicrobial resistance surveillance system by creating an accessible and informative website; and (v) international cooperation with multiplex networks not only with Asian countries but also with countries in other continents through hosting international symposiums and running an educational programme for developing countries through the Korea International Cooperation Agency.

## Conclusion

South Korea is currently experiencing many problems and challenges from AMR. Kor-GLASS is realiable, with scalability, and capability for better performance. Beyond monitoring AMR, this system is a useful tool for public health authorities to deal with AMR. Kor-GLASS’s successful AMR monitoring system has encouraged the South Korean government to establish a ‘one health’ approach for AMR in 2017 and to further develop the project by 2019 [22,23].
